# Population-Based Birth Cohort Studies in Epidemiology

**DOI:** 10.3390/ijerph17155276

**Published:** 2020-07-23

**Authors:** Cristina Canova, Anna Cantarutti

**Affiliations:** 1 Unit of Biostatistics, Epidemiology and Public Health, Department of Cardio-Thoraco-Vascular Sciences and Public Health, University of Padova, 35131 Padova, Italy; 2 Laboratory of Healthcare Research & Pharmacoepidemiology, Unit of Biostatistics, Epidemiology and Public Health, Department of Statistics and Quantitative Methods, University of Milano-Bicocca, 20126 Milan, Italy; anna.cantarutti@unimib.it

**Keywords:** birth cohort study, epidemiology, real-world data, record linkage, perinatal and postnatal exposure, environmental exposure, pharmacoepidemiology

## Abstract

Birth cohort studies are the most appropriate type of design to determine the causal relationship between potential risk factors during the prenatal or postnatal period and the health status of the newborn up to childhood and potentially adulthood. To date, there has been a growth in interest regarding observational population-based studies which are performed to provide answers to specific research questions for defined populations, for instance, assessing the exposure to environmental pollutants or drugs on the risk of developing a disease. Birth cohorts based on the recruitment and active follow-up of mothers and children allow the collection of biological material, and specific clinical and genetic information. However, they require a considerable amount of time and resources and, besides being usually of limited size, they are exposed to the risk of the loss of subjects to follow-up, with decreased statistical power and possible selection bias. For these reasons, linking the medical birth register with administrative health records for mothers and babies is increasingly being used in countries with a universal healthcare system, allowing researchers to identify large and unselected populations from birth, and to reconstruct relevant traits and care pathways of mothers and newborns. This Special Issue of the International Journal of Environmental Research and Public Health focuses on the current state of knowledge on perinatal and postnatal exposures and adverse pregnancy, maternal, fetal and neonatal outcomes through population-based birth cohort studies, with a specific focus on real-word data. The 12 accepted articles covered a wide range of themes that can be addressed specifically through birth cohort study design; however, only three were based on real word data with record-linkage to health administrative databases. In particular, two papers have addressed the topic of socioeconomic status considering several indicators both at the individual and contextual level. Two papers focused on inflammatory bowel diseases, both as an outcome of perinatal and antibiotic exposure in early life and as a condition associated with asthma, among children identified in a birth cohort based on a Regional Medical Birth Register. Three articles focused on medication use during pregnancy and its impact on maternal and fetal health. The effect of exposure to prenatal environmental risk factors on perinatal and childhood outcomes has been considered in two papers. Two papers analyzed ad hoc nationwide prospective birth cohorts set in Japan and UK. Finally, we included a systematic review with meta-analysis to evaluate the relation between growth restriction at birth and congenital heart defects. We think that this Special Issue may contribute to enriching the discussion of future challenges, opportunities, strengths and limitations for all research topics that can be investigated using a population-based birth cohort study design.

Birth cohort studies are the most appropriate type of design to determine the causal relation between potential risk factors during the prenatal or postnatal period and the health status of the newborn up to childhood and potentially adulthood. Since individuals are followed longitudinally across their life span from birth, or even from the intrauterine period, birth cohorts allow us to delineate associations between early exposures and subsequent outcomes [1]. Despite the difficulty of disentangling risk factors, the period of in utero development is one of the most critical windows during which adverse conditions and exposures may influence the growth and development of the fetus, as well as its postnatal developmental and behavioral outcomes [2]. Prenatal and early postnatal periods are therefore crucial to identify critical windows of susceptibility.

Population-based studies are defined as a group of individuals taken from the general population who share common characteristics, such as age, sex, or health conditions. These types of studies are performed to provide answers to specific research questions for defined populations, for instance assessing the response to a drug or the risk of developing a disease. The most delicate aspect is the selection of individuals included in the study: they should be representative of all individuals in the specific, a priori-defined population. Data collection in population-based studies relies both on linkage with available databases (e.g., hospital discharge records, death certificates, cancer registries, medical birth registries) and on procedures designed to ascertain study variables [3]. Computerized record linkage has clearly played, and continues to play, a relevant role in numerous birth cohort studies. This approach allows researchers to use retrospective follow-up which makes large studies feasible at a relatively low cost. Moreover, it allows researchers to identify health conditions through disease-specific case-identification algorithms that combine data deriving from one or more databases [4], and it can be of great use to monitor health conditions and exposures, especially pharmacological exposures, in a real-life context, without the costs, risks and ethical implications of a clinical trial. This approach can also contribute to provide insights and possible directions for future research to progress in the direction of phenomena observed at a population level. However, limits to these studies concern information relating to health behaviors and intermediate hospitalizations that are missing or under-reported.

Birth cohorts based on the prospective recruitment and active follow-up of mothers and children allow the collection of accurate information about exposures, outcomes and several covariates as well as biological material which is not usually included in retrospective studies. However, they require a considerable amount of time and resources and, besides being usually of limited size, they are exposed to the risk of a loss of subjects to follow-up, with decreased statistical power and possible selection bias. Furthermore, self-reporting is a common approach to acquire data in these studies with a risk of self-reporting bias [5].

Progressive technological and organizational improvements in recording, storing, and integrating healthcare data have raised interest in the use of real-world data to enhance the efficiency of research and to bridge evidentiary gaps between clinical research and practice. Real-world data derive from a variety of sources, including health administrative databases (HADs) and electronic health records. HADs are designed to collect information for administrative purposes, relating to all healthcare services provided by the National Health Service, such as dispensations of drug prescriptions, hospital discharge records, medical birth records and mortality records, exemptions from health-care copayments and so on. However, they are increasingly being used to examine features of healthcare delivery such as practice patterns, quality of care, safety and effectiveness of drugs, and other parameters that can be evaluated by means of epidemiological studies, as well as to support healthcare and policy decision-making [6]. Linking the medical birth registers with administrative health records for mothers and babies, first carried out in the Scandinavian countries [7,8], is increasingly being used in other countries with a universal healthcare system [9], allowing researchers to identify large and unselected populations from birth, and to reconstruct relevant traits and care pathways of mothers and newborns.

This Special Issue of the *International Journal of Environmental Research and Public Health (IJERPH)* focuses on the current state of knowledge on perinatal and postnatal exposures and adverse pregnancy, maternal, fetal and neonatal outcomes through population-based birth cohort studies, with a specific focus on real-word data. The 12 accepted articles covered a wide range of themes that can be addressed specifically through birth cohort study design; however, only three were based on real word data with record-linkage to HADs [10,11,12].

Two papers addressed the topic of indicators of socioeconomic disadvantage [10,13]. Evidence suggests that socioeconomic disadvantages in early-life can affect child health and have long-term effects also on adult health [14,15]. Assessing early-life socioeconomic status (SES) is also essential to control for confounding and modification effects in birth cohort studies. Spadea and colleagues established a network of population-based birth cohorts in five Italian cities, to study the role of exposure to air pollution and SES on birth outcomes [10]. They were able to individually link birth certificates, the municipal population register, hospital discharge records and small-scale models for residential air pollution exposures through geocoded residence addresses for all singleton livebirths from women aged 15–49 years at delivery, who were residents in the five cities during the period 2007–2013. The cohorts include all births in the area of interest and are not distorted by selection mechanisms or social desirability bias, such as birth cohorts based on voluntary enrolment and/or face-to-face interviews on socioeconomic characteristics. With the established network, the follow-up of mothers and newborns in the first years of life will be possible through linkage with hospitalizations, pharmaceutical prescriptions and outpatient services. Regarding SES, several indicators were considered, both at the individual level (maternal education, occupational status and citizenship) and contextual level (through a composite indicator of deprivation at the census block level). However, each single indicator captures different, likely correlated, dimensions of the child’s SES. As Pizzi and colleagues stated, the household’s disposable income is potentially one of the most important single indicators of the child’s SES, but it is underused because it is difficult to measure through questionnaires [13]. Moreover, comparing income across populations and studies might be complex, as different studies might collect different types of income and at different points in time. This is particularly relevant in the context of international collaborative studies, where it is essential to have harmonized comparable SES indicators. The authors therefore proposed a method for constructing a standardized and comparable cohort-specific household income indicator (“Equivalized Household Income Indicator (EHII)) for child SES to be used in European birth cohort studies, using external data from the pan-European surveys “European Union Statistics on Income and Living Conditions” (EUSILC) and internal data from four Italian and French birth cohorts. Being based on external data from the EUSILC surveys, which are conducted in several European countries using the same design and procedures, the EHII allows researchers to obtain a harmonized family income measure over different European populations, that can be used in the context of European collaborative studies [13].

Two papers focused on inflammatory bowel diseases, both as an outcome of perinatal and antibiotic exposure in early life and as a condition associated with asthma, among children identified in a birth cohort [11,12]. Both exposures and comorbidities were assessed by means of HADs, using a matched case-control design nested in a population-based birth cohort of more than 213,000 individuals born between 1989 and 2012, and resident in the Region of Friuli-Venezia Giulia (Italy).

Three articles focused on medication use during pregnancy [16,17,18], and its impact on maternal and fetal health, which are a growing public health concern. However, little information is available about the safety of most medications prescribed during this period, and clinicians often have to face the decision about whether or not to treat pregnant women based on the clinical setting of the mother. Some medicines may cause birth defects, pregnancy loss, prematurity, infant death, or developmental disabilities. The use of any medication including over-the-counter drugs, during pregnancy is estimated at 94%. However, studies have shown that less than 10% of medications approved from 1980 to 2010 have sufficient evidence to determine fetal risks deriving from in utero exposures [19].

Since pregnant women are always excluded from randomize controlled trials (RCT) due to ethical reason, it is very difficult to evaluate the safety of these drugs used during pregnancy. In this context, observational population-based cohort study designs offer an alternative to RCTs. The potential benefits of observational studies include the opportunity to evaluate a treatment’s effectiveness (i.e., results as seen in practice) rather than efficacy. Lower costs and difficulties that characterize observational studies allow researchers to enroll large numbers of patients, resulting in a greater statistical power. Therefore, it is possible to make inferences about differential effectiveness in subgroups of the population and generalize results to the overall population. However, beyond the limitations inherent in the observational nature of the approach, the lack of basilar information, and the consequent need of using proxies of the variables of interest, makes this approach particularly vulnerable to systematic uncertainty [6].

To conclude, medication use during pregnancy is very common. In the paper published in this issue, Lupatelli and colleagues mapped the patterns of medication use in pregnancy, as well as the extent and type of prescribed medications that are purposely avoided by pregnant women in Italy through a cross-sectional, web-based study [16]. According to the literature, they found a prevalence of total medication use during pregnancy of about 71%. Moreover, they did not find differences in estimates across Italy. Overall, 26.6% of women reported to have deliberately avoided a prescribed medication in pregnancy due to concerns about the safeguarding of maternal-child health. Also, Lutz and colleagues focused their attention on medication use among pregnant women with a high prevalence of self-medication in a birth cohort study in Brazil [17]. They found a prevalence of medication use during pregnancy that was higher than Lupatelli et al. (92.5%). Moreover, the same authors put their attention to another important field regarding medication use among women who breastfed their children [18]. It is widely recognized that there are many benefits of breastfeeding for both mother and child associated with lower morbidity and mortality. However, the use of medications by mothers may influence the success of breastfeeding. Lutz et al. did not find an association between weaning rates across the different breastfeeding safety categories of medications in women who were still breastfeeding, three months after birth. However, a limit to these results is that both the articles by Lupatelli et al. and Lutz et al. published in this Special Issue are based on self-reported information.

Exposure to prenatal environmental risk factors on perinatal and childhood outcomes has been considered in two papers [20,21]. Manduca and colleagues undertook hospital based surveillance of birth outcomes, collecting information on environmental exposures to potentially dangerous substances on about 15,000 women about to deliver in three different periods (from 2006 until 2019) in Gaza, Palestine, which has been the object of repeated severe military attacks since 2006 [20]. The second more methodological paper focused on prenatal exposure to airborne particles as a potential risk factor for infant neuropsychological development [21]. The aim of the study was to estimate the causal effect of prenatal exposure to high concentrations of airborne particles on children’s psychomotor and mental scores using a birth cohort from Gipuzkoa (Spain), and investigate possible unobserved confounding. They adopted a propensity score matching approach comparing the actual effect estimates with those obtained after adjusting for unobserved confounders based on simulations. Unmeasured confounding is an important limitation of observational studies and especially when new relationships are investigated, sensitivity analyses aimed to evaluate the robustness of the results to the omission of relevant factors in the analysis should become a standard [21].

The other two papers analyzed ad hoc nationwide prospective birth cohorts set in Japan (Japan Environment and Children’s Study [22]) and the UK (UK Millennium Cohort Study [23]). Both the topics covered in these studies (co-sleeping associated with less breathing difficulties and child development in relation to pet ownership at an early age) could not be assessed through retrospective real word data. The first manuscript used data from an ongoing nationwide prospective birth cohort study in Japan [22]. The recorded information is related to pregnant women who compiled the self-administered questionnaire between January 2011 and March 2014. Waynforth et al. used data drawn from the UK Millennium Cohort Study which recorded self-reported information from the mothers of 18,552 infants born from September 2000 to August 2001 in the UK [23].

Finally, Ghanchi and colleagues conducted a systematic review with meta-analysis to evaluate the relation between growth restriction at birth and congenital heart defects. The majority of studies included in the meta-analysis were population-based studies [24].

We, as Guest Editors, hope that this Special Issue may contribute to enriching the discussion of future challenges, opportunities, strengths and limitations for all research topics that can be investigated using a population-based birth cohort study design.
